# In Depth Analysis of Photovoltaic Performance of Chlorophyll Derivative-Based “All Solid-State” Dye-Sensitized Solar Cells

**DOI:** 10.3390/molecules25010198

**Published:** 2020-01-03

**Authors:** Michèle Chevrier, Alberto Fattori, Laurent Lasser, Clément Kotras, Clémence Rose, Michela Cangiotti, David Beljonne, Ahmad Mehdi, Mathieu Surin, Roberto Lazzaroni, Philippe Dubois, Maria Francesca Ottaviani, Sébastien Richeter, Johann Bouclé, Sébastien Clément

**Affiliations:** 1 ICGM, Univ. Montpellier, CNRS, ENSCM, CC1701, Place Eugène Bataillon, 34095 Montpellier, France; Michele.chevrier@stepaneurope.com (M.C.); clement.kotras@umontpellier.fr (C.K.); clemence.rose@umontpellier.fr (C.R.); ahmad.mehdi@umontpellier.fr (A.M.); 2 Service des Matériaux Polymères et Composites (SMPC), Centre d’Innovation et de Recherche en Matériaux et Polymères (CIRMAP), Université de Mons, 20 Place du Parc, 7000 Mons, Belgium; philippe.dubois@umons.ac.be; 3 Department of Pure and Applied Sciences (DiSPeA), University of Urbino, 61029 Urbino, Italy; alberto.fattori@uniurb.it (A.F.); michela.cangiotti@uniurb.it (M.C.); maria.ottaviani@uniurb.it (M.F.O.); 4 Laboratory for Chemistry of Novel Materials, CIRMAP, University of Mons UMONS, 20 Place du Parc, 7000 Mons, Belgium; laurent.lasser@umons.ac.be (L.L.); david.beljonne@umons.ac.be (D.B.); mathieu.surin@umons.ac.be (M.S.); roberto.lazzaroni@umons.ac.be (R.L.); 5 CNRS, Univ. Limoges, XLIM, UMR 7252, F-87000 Limoges, France

**Keywords:** solid state dye sensitized solar cells, spirulina, chlorophyll, anchoring groups, EPR

## Abstract

Chlorophyll *a* derivatives were integrated in “all solid-state” dye sensitized solar cells (DSSCs) with a mesoporous TiO_2_ electrode and 2′,2′,7,7′-tetrakis[*N*,*N*-di(4-methoxyphenyl)amino]-9,9′-spirobifluorene as the hole-transport material. Despite modest power conversion efficiencies (PCEs) between 0.26% and 0.55% achieved for these chlorin dyes, a systematic investigation was carried out in order to elucidate their main limitations. To provide a comprehensive understanding of the parameters (structure, nature of the anchoring group, adsorption …) and their relationship with the PCEs, density functional theory (DFT) calculations, optical and photovoltaic studies and electron paramagnetic resonance analysis exploiting the 4-carboxy-TEMPO spin probe were combined. The recombination kinetics, the frontier molecular orbitals of these DSSCs and the adsorption efficiency onto the TiO_2_ surface were found to be the key parameters that govern their photovoltaic response.

## 1. Introduction

Fossil fuel resource exhaustion, increasing energy demands and environmental problems have triggered an intensification of research for sustainable energy sources, which lead to the development of photovoltaic devices, fuel cells, wind turbines, etc. Photovoltaic conversion of solar energy is considered to be one of the most significant ways of addressing the growing global energy crisis [1]. In this context, utilizing biomass resources for the conversion of solar energy has been receiving increasing attention from industry, academia and governments and will become a major challenge of our future [2,3,4]. Biomass has a great potential as a renewable feedstock for producing solar energy [5,6]. Indeed, protein-pigment complexes in plants, green algae, and cyanobacteria convert solar energy into chemical energy through oxygenic photosynthesis with high efficiency [7,8,9]. Calvin showed through a model based on a photosynthetic electron transport system containing membranes, carotenoids and pigments that electric energy can be obtained by conversion of sunlight [10]. A similar approach based on an artificial photosynthesis led in 1991 to dye-sensitized solar cells (DSSCs, also known as Grätzel cells) [11]. A DSSC consists of a mesoporous nanocrystalline *n*-type semiconductor (typically TiO_2_) sensitized with a dye, deposited onto an anode and immersed in redox active electrolyte (generally triiodide/iodide), completed by a counter-electrode (cathode) [12]. Upon illumination, dye molecules anchored onto the TiO_2_ surface absorb incoming photons, allowing the photoinduced electron-transfer from their excited state into the TiO_2_ conduction band. Then, the electrons are transferred to the counter electrode, thus creating a current. The redox electrolyte reduces the oxidized dye molecules back to their ground state to enable continuous electron production. Even if power conversion efficiencies (PCEs) above 13% have been reached for liquid-electrolyte DSSCs [13,14], improvements are still required in order to be commercially viable.

First, there is a crucial need for solid-state approaches since the use of liquid electrolytes is a major hurdle for commercialization by industry due to the corrosive nature of the commonly used I_3_^−^/I^−^ redox shuttle [15,16]. DSSCs incorporating solid-state hole transport materials (HTMs) have been actively investigated [17,18,19], and decent efficiencies close to 12% have been already achieved by molecular and inorganic HTMs combined with state-of-the-art organic dyes [20,21]. Additionally, solid-state DSSCs (ssDSSCs) led to a major breakthrough by using hybrid lead halide perovskite sensitizers such as CH_3_NH_3_PbI_x_Cl_3-x_, enabling the advent of perovskite solar cells with efficiencies now above 25% [22]. However, important issues concerning the device stability and the toxicity of the components still remain.

Secondly, it is highly required to use efficient and cost-effective dyes in place of expensive and scarce ruthenium complexes [23,24] or elaborated organic dyes requiring numerous synthetic steps [15,25]. In contrast, natural organic dyes and their derivatives are promising candidates for developing DSSCs due to their low cost, abundance and non-toxicity [5]. An important biomass resource developed in the south of France is spirulina, a green algae mainly used as food supplement. One of the most valuable compound that can be extracted from spirulina is chlorophyll *a*, (Chl *a*—12 g/kg) [26] a photo- and electroactive chlorin-type macrocycle involved in photosynthesis, because it efficiently absorbs light in the 400–450 nm and the 600–700 nm regions and up to 800 nm depending of its aggregation state [27]. The use of chlorophyll derivatives as sensitizers has been investigated in liquid electrolyte-based DSSCs leading to energy-to-electricity conversion efficiency (η) up to around 8% [28,29,30]. Concerning solid-state DSSC, there are only a few reports in the literature describing the use of chlorophyll *a* derivatives as dye [31,32,33].

**Figure 1:**
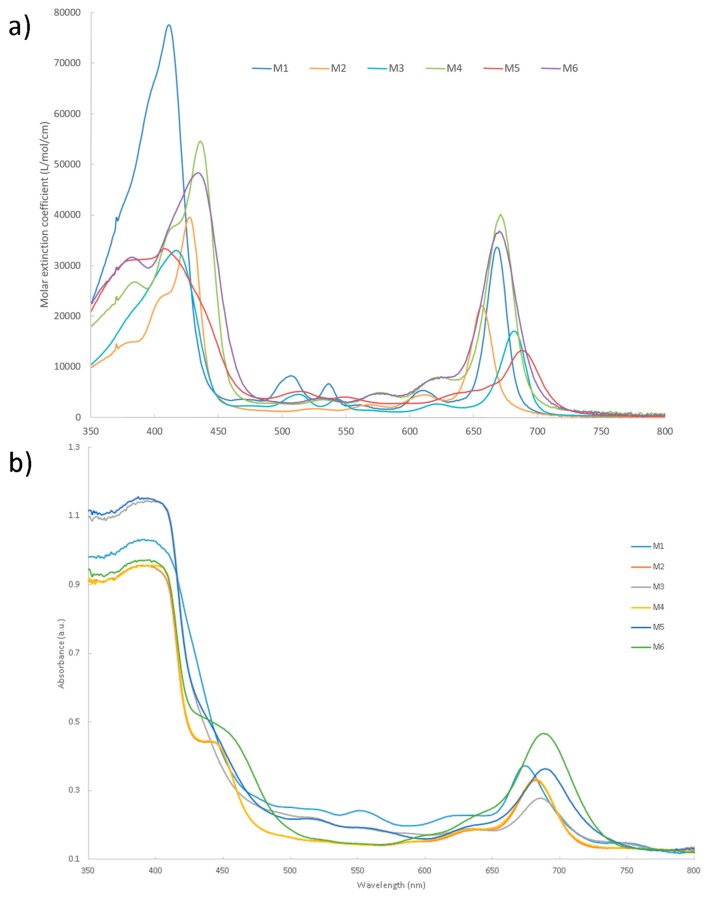
(**a**) UV-Visible absorption spectra of chlorins **M1**–**M6** in THF and (**b**) grafted onto the TiO_2_ surface.

Herein, we report the preparation of all-solid state DSSCs using a series of Chl *a* derivatives (Figure 1) as sensitizers with different anchoring groups to graft to the TiO_2_ surface and 2′,2′,7,7′-tetrakis[*N*,*N*-di(4-methoxyphenyl)amino]-9,9′-spirobifluorene (spiro-OMeTAD) as HTM. The choice of spiro-OMeTAD HTM was motivated by its facile implementation and high performance rendering it particularly suitable for testing DSSCs. To elucidate the relationship between the molecular structure of the dye and the photovoltaic efficiency, UV-Vis absorption spectroscopy, density functional theory (DFT) calculations, incident photon-to-current efficiency (IPCE), transient photovoltage decay measurements and EPR analysis exploiting the spin probe 4-carboxy-2,2,6,6-tetramethylpiperidine 1-oxyl (4-carboxy-TEMPO, indicated as **4C-T**) have been carried out.

## 2. Results

### 2.1. Synthesis of the Dyes Derived from Chlorophyll a

**Scheme 1:**
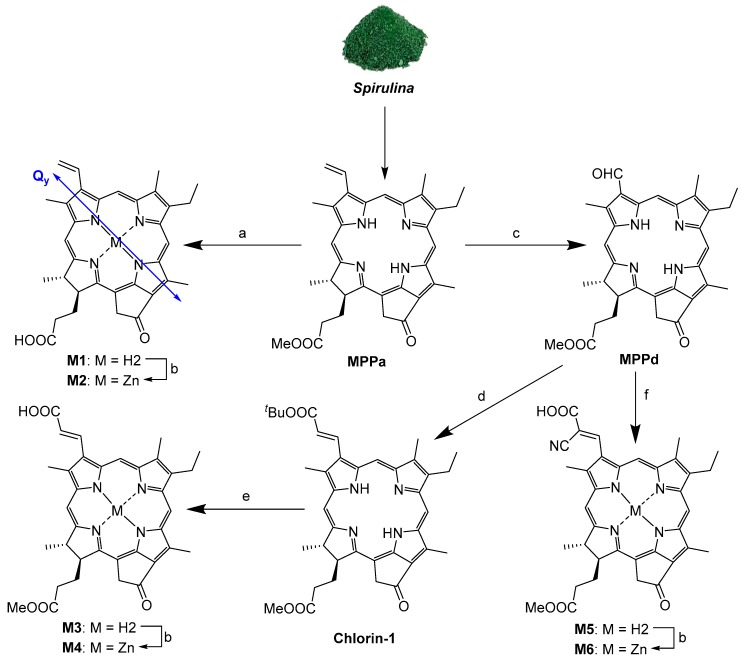
Synthesis of chlorins **M1**–**M6** from **MPPa**: (**a**) HCl, acetone; (**b**) Zn(OAc)_2_·2H_2_O, MeOH/THF; (**c)** OsO_4_, NaIO_4_, dioxane-AcOH-water; (**d**) Ph_3_P=CHCOO*^t^*Bu, CH_2_Cl_2_; (**e**) CF_3_COOH; and (**f**) NCCH_2_COOH, piperidine-THF.

The chemical structures of chlorophyll *a*-based dye sensitizers depicted in Scheme 1 were obtained from methyl pyropheophorbide-*a* (**MPPa**). **MPPa** was prepared in four steps: (i) extraction of Chl *a* from spirulina biomass with ethanol, (ii) demetallation of Chl *a*, (iii) acid-catalyzed transesterification of the phytyl chain in methanol, and (iv) decarboxylation of the ester moiety located on the 13-position with 2,4,6-collidine [34,35]. Three families of corresponding free-base and zinc chlorins can be distinguished depending on the localization of the anchoring group. All the chlorins have one carboxylic acid for grafting onto the TiO_2_ surface. Chlorins **M1**–**M2** exhibit a non-conjugated alkyl anchoring group while the others possess either a conjugated vinyl anchoring group (**M3**–**M4**) or a cyanoacrylic group along the Q_y_ axis of the chlorin skeleton (**M5**–**M6**).

**M1** was synthesized through the acid hydrolysis of **MPPa** with hydrochloric acid in acetone. Methyl pyropheophorbide-*d* (**MPPd**) was prepared by cleavage of the alkene in the presence of osmium tetroxide (OsO_4_) and sodium periodate (NaIO_4_) [35]. Trans-3^2^-carboxy-pyropheophorbide-*a* (**M3**) was synthesized in two steps: (a) a Wittig reaction with **MPPd** and (b) the hydrolysis with TFA of the corresponding **Chlorin-1**. A Knoevenagel reaction of **MPPd** with cyanoacetic acid in piperidine/THF yielded chlorin **M5**. Finally, metalation reactions of the free base chlorins **M1**, **M3**, and **M5** with Zn(OAc)_2_ in MeOH/THF afforded the corresponding zinc complexes **M2**, **M4**, and **M6**.

### 2.2. Optical Properties of Chlorin Derivatives

The UV-Visible absorption spectra of chlorins **M1**–**M6** both in THF and grafted on the TiO_2_ surface through carboxylate bonding are presented in Figure 1. Table 1 summarizes their characteristics in THF and on TiO_2_. The shape of the UV-Visible absorption spectra in THF are relatively similar, with five observed bands when going from blue to red: Soret, Q_x_ (0,1), Q_x_ (0,0), Q_y_(0,1), and Q_y_(0,0). The Q_y_ peaks position of the vinyl-substituted chlorins **M3**–**M6** are redshifted by about 10–20 nm in comparison with chlorins **M1**–**M2** due to the introduction of electron-withdrawing groups on the vinyl moiety at the C3 position. The bathochromic shifts of the Q_y_ peaks of chlorins **M5**–**M6** are larger than those observed for **M3** (7–15 nm) and **M4** (1–4 nm) because of the higher electron-withdrawing ability of the cyano group. Upon zinc metalation, the Soret absorption bands are redshifted (chlorins **M2**, **M4**, and **M6**), implying an increase of the conjugated π-electron delocalization along X axis while the hypsochromic shift of the Q_y_ indicates a decrease of the extent of conjugated π-electron delocalization along Y axis [36].

The binding of the chlorin dyes onto the TiO_2_ surface leads to a broadening of the absorption bands, probably due to π stacking interactions between chlorin dye molecules, as extensively studied in previous investigations [37]. The comparison of the chlorin absorption spectra in THF solutions as the monomeric species (Figure 1a) and on TiO_2_ as aggregates also reveal that the Q_Y_ bands shift to longer wavelengths suggesting the formation of *J* aggregates [31].

### 2.3. Photovoltaic Performance of Chlorin Derivatives

#### 2.3.1. Influence of the Co-Adsorbent

The π stacking of dyes when grafted onto the TiO_2_ surface was evidenced from the UV-Visible absorption spectra, as discussed above. Such aggregation is known to affect the photoelectron injection efficiency and thus, the overall conversion efficiency [38,39]. A typical way to lower the dye aggregation at the semiconductor interface consists in using co-adsorbents such as chenodeoxycholic acid (CDCA), the most commonly used additive in DSSC preparation [40,41,42]. Indeed, its strong binding to the TiO_2_ surface partly displaces dye molecules, reducing their self-aggregation. Nevertheless, the addition of co-adsorbent molecules also reduces the dye loading and thus, photon harvesting. It is therefore crucial to judiciously adjust the amount of co-adsorbent used during the impregnation. To optimize the loading of the co-adsorbent in the dye solution, the impregnation was performed for 15 h using different concentrations of CDCA (from 0 to 3 mM) in a 0.3 mM THF solution of the dye. The choice of THF was motivated by the good solubility of the free-base and zinc-metalated chlorin in that solvent.

**Figure 2:**
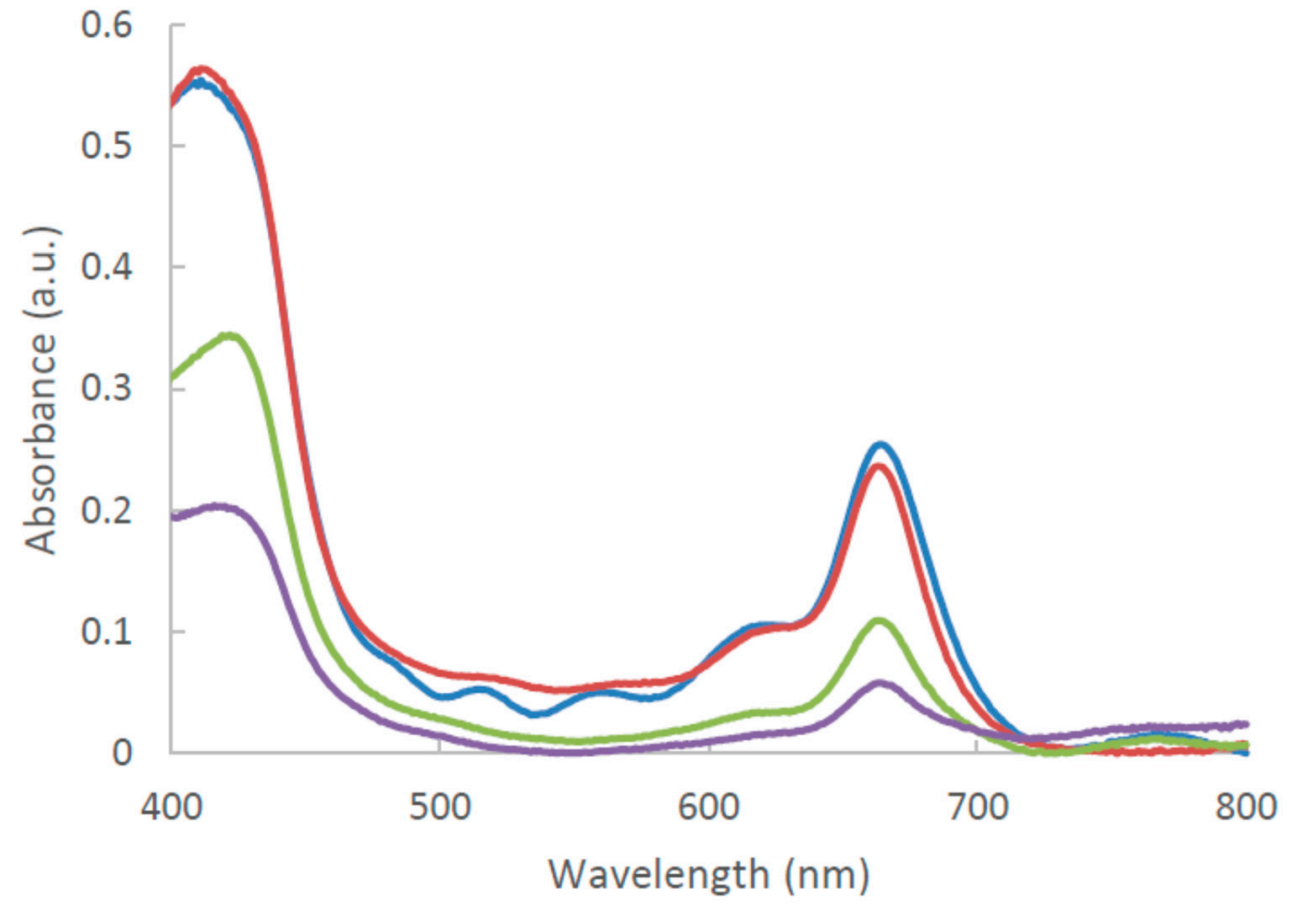
UV-Visible absorption spectra of TiO_2_ films impregnated with **M2** (0.3 mM in THF) and increasing co-adsorbent concentration (0 mM: blue line; 0.3 mM: red line; 1.5 mM: green line, and 3 mM: purple line in THF).

To determine the amount of dye **M2** grafted onto the TiO_2_ surface as a function of the concentration of co-adsorbent, UV-Visible absorption measurements were performed (Figure 2). Dye **M2** grafted from a 0.3 mM solution shows an absorbance of 0.55 for the Soret absorption band at around 420 nm and 0.25 for the lower energy Q band at around 665 nm. This value dramatically decreased when the concentration of co-adsorbent was increased up to 3 mM and this spectral evolution is in good agreement with previous studies reported in the literature [43,44]. Indeed, the co-adsorbent tends to decrease the adsorption of dye **M2** via a competitive anchoring process, thereby decreasing the dye concentration and thus, the dye aggregation onto the TiO_2_ surface.

**Figure 3:**
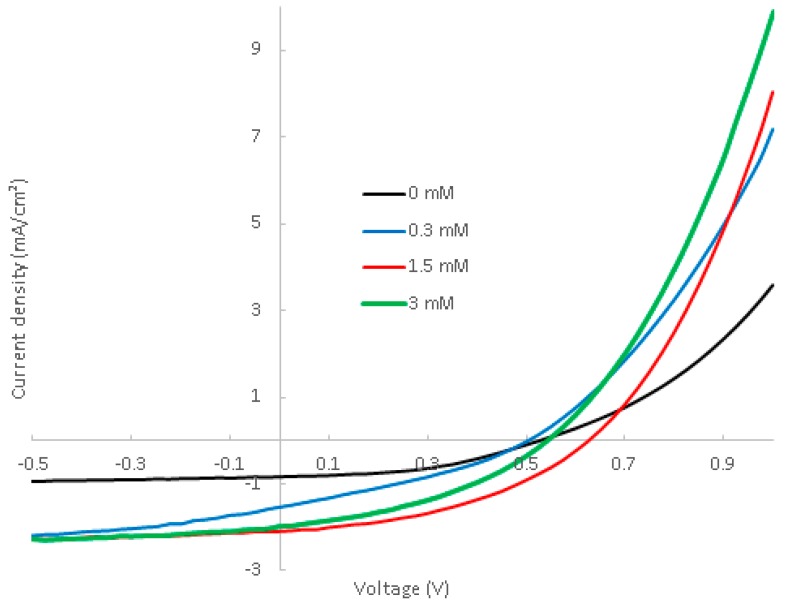
Current–voltage characteristics of solid-state dye sensitized solar cells (ssDSSCs) sensitized with dye **M2** depending on the co-adsorbent concentration.

The current density–voltage (J–V) characteristics of the corresponding devices were then measured and are depicted in Figure 3. The extracted photovoltaic parameters are presented in Table 2. To evaluate the efficiency of our fabrication process and thus, the accuracy of the collected data, a solar cell based on the well-known organic dye, **D102**, was used as reference [45,46].

The performance obtained for the reference cell is in agreement with those previously reported in the literature [45,46]. Modest photovoltaic performances are observed for dye **M2** compared to liquid DSSCs, where the short-circuit current density (*J*_SC_) was found to be reduced by a factor of about 10. This finding could mainly be attributed to the reduction in the electrode thickness for solid-state devices, leading to a lower light harvesting efficiency of the active layer. The devices where the TiO_2_ electrode was sensitized with the dye **M2** alone, gave photovoltaic efficiencies of 0.19%. When **M2** was combined with **CDCA**, the PCE increased up to 0.56% for the 1:5 molar ratio. Further increase in co-adsorbent to the 1:10 molar ratio caused a decrease in efficiency (η = 0.42%), attributed to the dye loading reduction.

**Figure 4:**
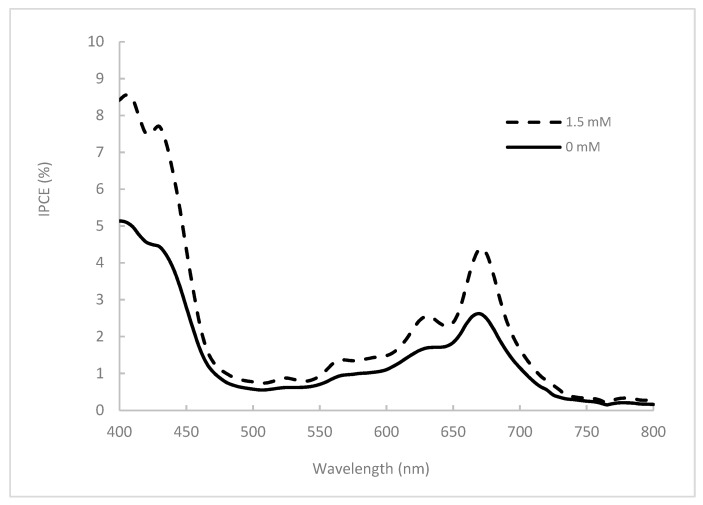
Incident photon-to-current efficiency (IPCE) spectra of all-solid state DSSCs sensitized with **M2** in the presence/absence of co-adsorbent.

The incident photon-to-current efficiency (IPCE) recorded for the above mentioned ssDSSCs indicates that the photocurrent response of **M2**-based DSSC impregnated with 1.5 mM of co-adsorbent exceeds 4% for the last Q band at 675 nm, which is higher than the response without co-adsorbent (2% at the same wavelength; Figure 4). The estimated photocurrent, obtained by integrating the IPCE spectra over the AM1.5G solar spectrum, was consistent with the photocurrent measured under solar simulator and reported in Table 2. Adding 1.5 mM of co-adsorbent for the impregnation enabled to double the current density value from 0.60 to 1.30 mA·cm^−2^ (0.73 vs. 1.73 mA·cm^−2^ in Table 2, respectively). The increased quantum yield was ascribed to the reduction of dye aggregation in the presence of **CDCA** as co-adsorbent, which minimized the intermolecular energy transfer and increased the electron injection efficiency. This effect compensated the loss of light harvesting caused by the lower dye content of the TiO_2_ photoanode with **CDCA** as co-adsorbent and led to the improvement of the current density. Based on this result, a concentration of 1.5 mM of co-adsorbent was chosen for the preparation of ssDSSCs since it offered the best compromise between light harvesting and power conversion efficiency.

#### 2.3.2. Photovoltaic Measurements for the Other Chlorin Derivatives

**Figure 5:**
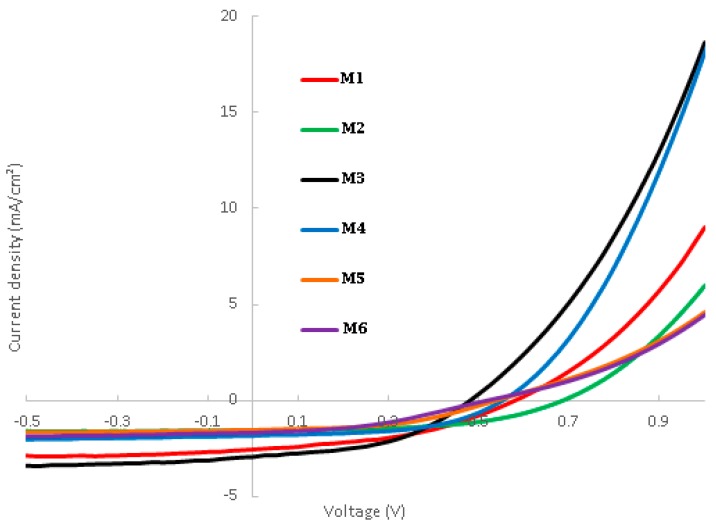
Current-voltage characteristics of ssDSSCs sensitized with chlorins **M1**–**M6** under illumination.

Figure 5 shows the J–V characteristics of the ssDSSCs prepared from dyes **M1** to **M6** (0.3 mM in THF) and **CDCA** co-adsorbent (1.5 mM in THF). The corresponding photovoltaic data are listed in Table 3.

Compared to the **D102** performances, those obtained with chlorin dyes were low (Table 3). Nevertheless, only a few publications have described the fabrication of ssDSSCs with chlorophyll derivatives and the values obtained here are in line with those reported [31]. They were even better than the performances obtained in some liquid-DSSCs with other chlorophyll derivatives [47,48,49,50]. ssDSSCs based on dye **M3** gave the highest PCE (0.67%) due to the highest short-circuit current (*J*_SC_), while the open-circuit photovoltage (V_OC_) and fill factors (FF) were found to be similar to other chlorin dyes, except for **M2**, which showed the highest V_OC_ value. In terms of grafting groups, the PCE values for ssDSSCs based on chlorin dyes exceed 0.26% in the following order: acrylic acid > alkyl acid > cyanoacrylic acid. The introduction of cyano electron-withdrawing groups in chlorins **M5**–**M6** indeed strongly decreased the *J*_SC_ values, resulting in the ssDSSCs with the lower performances. This trend has been already observed when such chlorin dyes were used in liquid DSSCs [51]. Nevertheless, the difference observed in ssDSSCs was not as dramatic as in liquid DSSCs. For instance, the presence of cyano groups on the free base chlorin **M3** drastically decreased the PCE in liquid DSSCs by around 75% while the decrease represents only 37% in ssDSSCs.

#### 2.3.3. Recombination Kinetics

**Figure 6:**
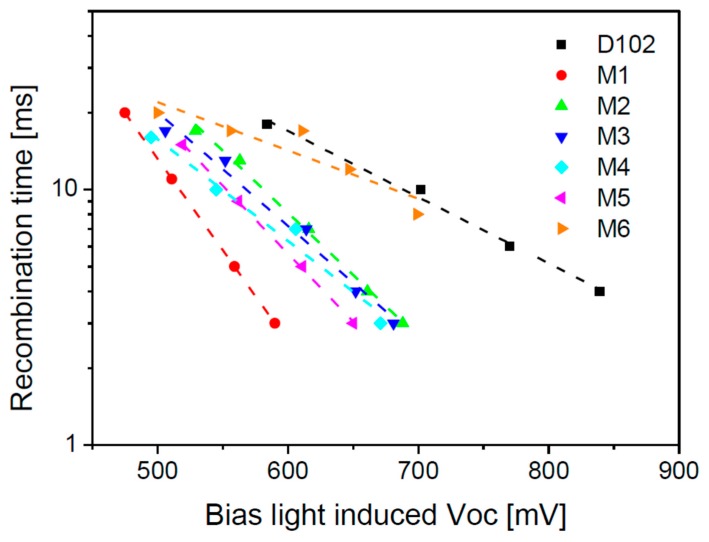
Electron recombination lifetime as a function of Voc in chlorin- and **D102** based ssDSSCs.

To examine the recombination kinetics of chlorin-based ssDSSCs, transient photovoltage decay measurements (TPV) measurements were carried out and compared to **D102**. Since the porous electrode and the anchoring groups of the dyes are quite similar (i.e., same fabrication process and only one carboxylic anchoring function on each dye), TPV measurements reflect the direct influence of the dye on the TiO_2_/dye/spiro-OMeTAD interface. The observed differences in recombination kinetics could mainly be attributed to the chemical nature and/or steric conformation of the dye at the TiO_2_ surface as well as the contribution from the dye/spiro-OMeTAD interface. The observed difference in slope between **D102** and the chlorin dye sensitizers indicates that different recombination mechanisms took place in ssDSSCs sensitized with these compounds (Figure 6). The recombinations for chlorin-sensitized solar cells appeared to be faster than that with **D102**, thus leading to a V_OC_ loss. This result was coherent with the photovoltaic parameters obtained under illumination. Indeed, chlorin-based ssDSSCs exhibited V_OC_ values around 550 mV, except for **M2** whereas that based on the **D102** dye sensitizer led to a V_OC_ of 760 mV.

Besides, chlorin dyes **M3** and **M5** show an enhanced electron lifetime in comparison with the **M1** analog. Electron lifetime associated with dye **M3** was also slightly larger than that of dye **M5**, which suggests acrylic acid anchoring group was more favorable than cyanoacrylic acid anchoring group to decrease the charge recombination at the TiO_2_/electrolyte interface. However, the performances of ssDSSCs based on **M5** were lower than those based on **M1**. Since the dye **M1** contains a non-conjugated anchoring group, **M1** may lie rather flat on the TiO_2_ surface due the larger flexibility of the alkyl spacer, whereas the more rigid conjugated spacer of **M3** and **M5** probably favors tilted or up-right adsorption. Finally, the influence of the zinc metal center was more difficult to interpret: on one hand, **M2** presented a significant increase in electron lifetime compared to **M1**; on the other hand, **M3** and **M4** were in the same range. These findings were coherent with the photovoltaic parameters obtained under illumination. As discussed above, **M2** exhibited a higher Voc than **M1** while the Voc was approximately the same for **M3** and **M4**.

### 2.4. DFT Calculations

**Figure 7:**
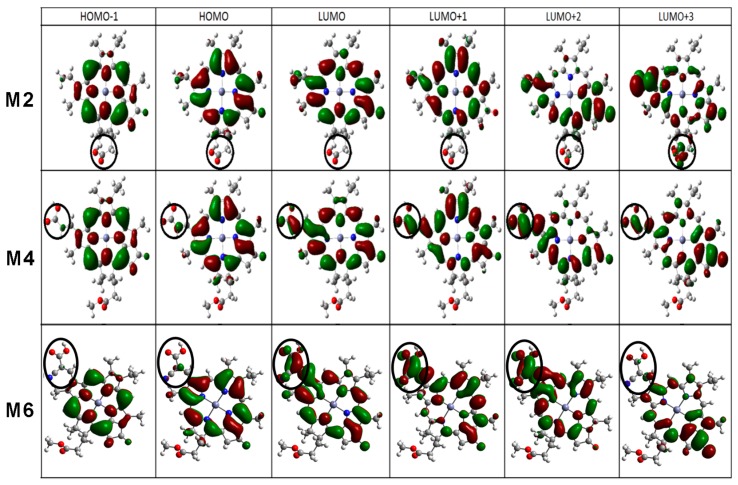
HOMO-1, HOMO, LUMO, LUMO + 1, LUMO + 2, and LUMO + 3 molecular orbitals for **M2** (top) and **M4** (middle) and **M6** (bottom) dyes. The anchoring groups are circled in black.

To elucidate the relationship between the nature of the anchoring group and the photovoltaic performances observed, density functional theory (DFT) calculations were performed. The calculations show that all the LUMO + n orbitals had a significant contribution from the acrylic group for **M4** and from the cyanoacrylic acid group for **M6** while only the LUMO + 3 orbital covered the alkyl carboxylic group for **M2** (Figure 7). This is critically important since the orbital overlap between the LUMO + n of the dye and the TiO_2_ conduction band edge should be as large as possible in order to maximize the electron transfer probability. **M4** and **M6** dyes exhibited similar π-electron systems leading to similar electron density on the π-conjugated anchoring group for LUMO, LUMO + 1, and LUMO + 2 orbitals.

**Figure 8:**
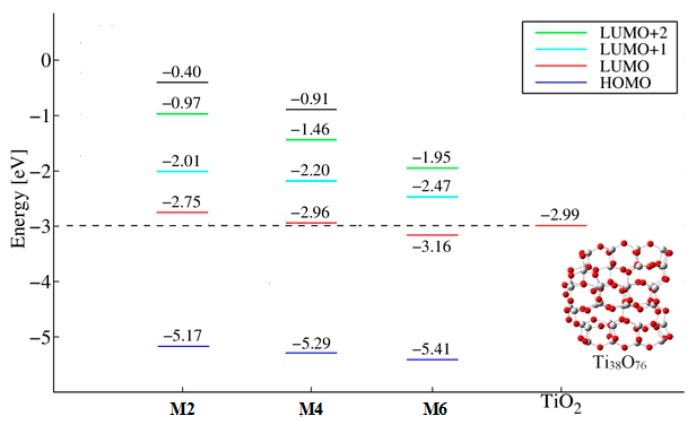
LUMO, LUMO + 1, and LUMO + 2 energy levels of chlorin dyes **M2**, **M4**, and **M6**. The values for the conduction band edge of the semiconductor calculated from a Ti_38_O_76_ cluster is added for comparison (dashed line).

However, introducing the electron-withdrawing cyano group on the acrylic acid anchoring results in a strong stabilization of the unoccupied orbitals (Figure 8). Indeed, the **M6** LUMO moved down below the TiO_2_ conduction band edge energy level, no longer allowing exergonic electron injection to TiO_2_ upon (HOMO → LUMO) optical transition, thus, explaining the decrease in PCE of **M6** compared to **M4**. The energy levels of HOMO and LUMO of **M2**, **M4**, and **M6** dyes are in the same range as those previously reported in the literature [31,33,52].

### 2.5. Dye Adsorption

To get insight into the relationship between the observed performances and the dye structure, the dye adsorption of free-base chlorins **M1**, **M3**, and **M5** was investigated by the use of electron paramagnetic resonance spectroscopy (EPR). This experimental technique allowed us to compare the cells performance with the dye adsorption on the TiO_2_ thanks to a paramagnetic radical probe 4-carboxy TEMPO (coded as **4C-T**). This molecule grafts onto the TiO_2_ surface without perturbing the system and is able to monitor its molecular surrounding through spin–spin interactions. As described more in depth elsewhere [53], the main parameters extracted from the spectra are as follows: (i) A_ii_, which is the component of the hyperfine coupling tensor between the electronic and nuclear spin that provides a measurement of the probe environmental polarity; (ii) the correlation time for the radical rotational diffusion motion, τ, that is related to the radical environmental microviscosity, which, in turn, increases due to interactions between the radical and its environment; (iii) the intrinsic line width, its increment in slow motion conditions being due to dipole−dipole spin−spin interactions (through this parameter, interactive close sites can be identified); and (iv) the Heisenberg exchange frequency, Wex, which increases when radicals swap each other the unpaired electrons due to collisions in a fluid medium (this parameter is related to a high radical local concentration on the TiO_2_ surface). In many cases, the EPR spectra are constituted by two well-identifiable components characterized by probes at different polarity and mobility. In order to extract the two components and to evaluate the relative percentages, experimental spectra were subtracted from each other and then the components were separately simulated and doubly integrated to evaluate the relative percentages. If subtraction of experimental spectra cannot be performed, one component is simulated and the other one is determined by subtraction of the simulated spectrum from the total one. Afterwards, the second component is also simulated. The accuracy for all the parameters in the EPR analysis was between 2% and 3%, based on the fitting between the experimental and the computed line shapes, slightly increasing if line broadening occurs.

**Figure 9:**
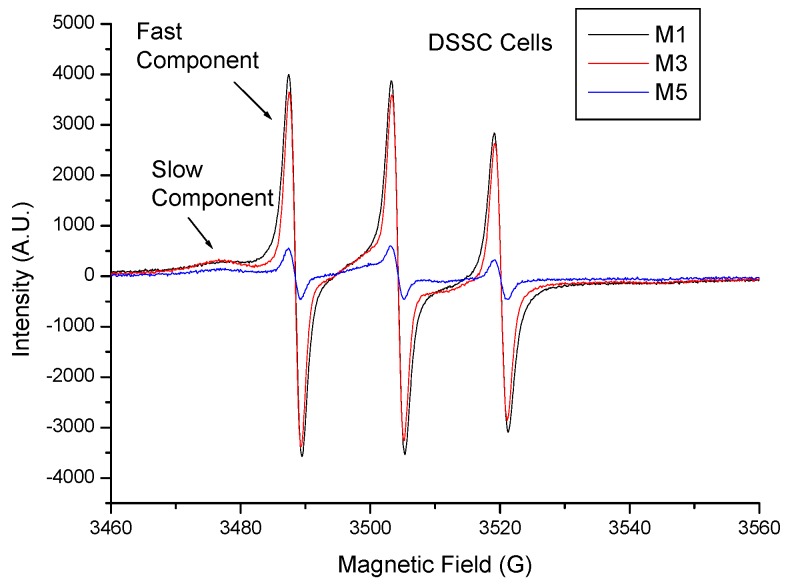
Electron paramagnetic resonance spectroscopy (EPR) spectra of DSSCs sensitized with the chlorine dyes **M1** (black line), **M3** (red line) and **M5** (blue line). The relative fast component and free component of the spectra are shown in the graph.

Figure 9 shows the EPR spectra obtained for DSSCs built with the different dyes **M1**, **M3**, and **M5** and containing the spin probe **4C-T**. These spectra were considered good if reproducible at least for three different cells built in the same experimental conditions.

From Figure 9, we could determine that: (i) the intensity significantly decreased for DSSC-**M5** with respect to the other DSSC-dyes. The intensity was evaluated by double integrating the spectra (Table 1). We also noticed that the intensity largely decreased when compared to devices containing only the spin probe (results not shown) and (ii) the spectra were constituted by two components whose main features are indicated in the figure. These two components were extracted and quantified (Table 4).

**Figure 10:**
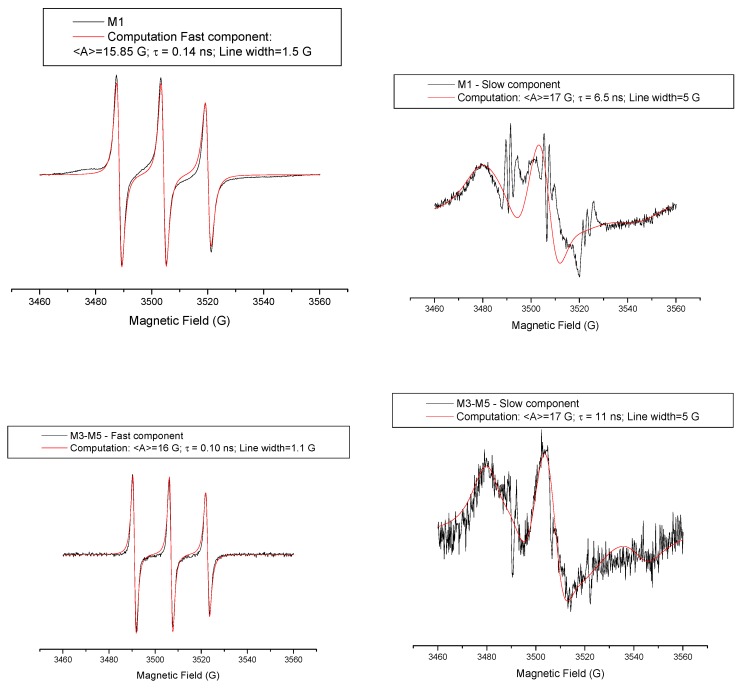
Experimental and computed spectra (extracted slow and fast component) for the probe **4C-T** interacting with the dyes **M1**, **M3**, and **M5**.

Each component was then computed as shown in Figure 10 for the different cells. The figure also shows the main parameters used for the computation, which are the <A> value (=(A_xx_ + A_yy_ + A_zz_)/3) measuring the polarity, the correlation time for the rotational motion, τ, measuring the interactions on the basis of the slowing down of mobility, and the line width, measuring the local concentration of the radicals. Based on the correlation time for the rotational motion, τ, obtained from the computations, the two components were termed “fast” and “slow”, since they originated from spin probes quite free to move at the interface and interacting with the TiO_2_ surface, respectively.

The low intensity of the spectrum of DSSC-**M5** if compared to the other DSSCs clarified the behavior of these dyes: **M5** was poorly adsorbed, but the probes and **M5** preferentially interacted with the surface (slow component) and only a small amount of dyes remained at the interface (free component). In other words, the total amount of adsorbed dye **M5** as well as the amount of interacting probes + **M5**-dyes were small. That amount was obviously smaller than the amount of probes and dye **M3** adsorbed at the TiO_2_ surface. Considering the slow component, it was the same for **M5** and **M3** corresponding to strongly interacting dyes at polar, quite close sites. Conversely, the fast component shows a high mobility and the environment was less polar than that of the slow component. These results also explained why dye **M3** exhibited better performances than **M5**. **M1** was the less interacting dye, not only marked by the lowest percentage of slow (interacting) component compared to the other dyes, but also because the slow component is slower (lower τ) in comparison with the other dyes. It is also interesting to note that the fast component for **M1** shows a slightly lower mobility, lower polarity and higher line width compared to the fast component from the other dyes. This indicates a higher local concentration of **M1** dyes in solution, in line with a lower adsorption at the TiO_2_ surface.

## 3. Materials and Methods

### 3.1. Instrumentation and Methods

Spirulina powder was provided from Phyco-Biotech (Montpellier, France). Titania paste (99% anatase), titanium(IV) chloride (99,9%), chenodeoxycholic acid (96%), bis(trifluoromethane)sulfonimide lithium salt (99%), 4-*tert*-butylpyridine (98%) were purchased from Sigma-Aldrich Chemie S.a.r.l. (Saint-Quentin Fallavie, France). Spiro-OMeTAD and FTO-coated conducting glass substrates were purchased from Solaronix SA (Aubonne, Switzerland). Reactions were performed under argon using oven-dried glassware and Schlenk techniques. Dry solvents were obtained by using a solvent purification system, PureSolve MD5 purchased from Inert Technology (Amesbury, MA, USA). ^1^H and ^13^C{^1^H} NMR spectra were recorded either on a Bruker Avance-300, 400, or 600 spectrometers (Bruker, Billerica, MA, USA) for each synthetic intermediates and were calibrated to tetramethylsilane (TMS) on the basis of the relative chemical shift of the solvent as an internal standard. Chemical shifts (δ) are expressed in parts per million (ppm) and coupling constants (*J*) are expressed in Hertz. Abbreviations used for ^1^H-NMR spectra are as follow: s singlet, d doublet, and m multiplet. Mass spectra (MS) were recorded on MicroTOF QII t and RAPIFLEX instruments (Bruker France SAS, Marne la Vallée, France) in the positive mode for ESI and MALDI-ToF, respectively. UV-Visible absorption spectra were obtained at room temperature on a Shimadzu UV2401 PC (Shimadzu Corporation, Kyoto, Japan) and on a SAFAS D.E.S. PC UV/Vis scanning spectrometers (Safas, Monaco).

### 3.2. Synthesis of Dyes ***M1***, ***M3***, and ***M5***

Methyl pheophorbide-*a* [34], methyl pyropheophorbide-*a* (**MPPa**) [34], methyl pyropheophorbide-*d* (**MPPd**) [35], **M1 [28]**, **M2 [54]**, **M3 [28]**, **M4 [55]**, **M5 [56]**, and **M6 [55]** were synthesized as reported in the literature (see Scheme 1 for the synthetic route).

**M1**: Methyl pyropheophorbide-*a* (150 mg, 2.74 mmol) was dissolved in acetone (15 mL) and concentrated hydrochloric acid (HCl 37%, 5 mL) was added slowly. After 30 min under stirring, the mixture was poured into water and extracted with CH_2_Cl_2_. The organic layer was then washed with a saturated solution of NaHCO_3_ and water until neutrality. The organic layer was dried over MgSO_4_, filtered, and concentrated under reduced pressure. The obtained solid was then recrystallized from a CH_2_Cl_2_/*n*-pentane mixture to give the title compound as a dark solid (129 mg, 88%). ^1^H-NMR (300 MHz, CDCl_3_): δ = 9.50, 9.39, 8.58 (s, each 1H, 5-, 10-, and, 20-H), 7.98 (dd, ^3^*J*_H-H_ = 17.8, 11.5 Hz, 1H, 3^1^-H), 6.22 (m, 2H, 3^2^-CH), 5.18 (m, 2H, 13^2^-CH_2_), 4.49, 4.41 (m, each 1H, 17- and 18-H), 3.64 (m, 3H, 8^1^-CH_2_ and 12^1^-CH_3_), 3.40 (2-CH_3_), 3.23 (s, 3H, 7-CH_3_), 2.70–2.30 (m, 4H, 17^1^ and 17^2^-CH_2_), 1.82 (d, ^3^*J*_H-H_ = 7.1 Hz, 3H, 18^1^-CH_3_), 1.68 (t, ^3^*J*_H-H_ = 7.5 Hz, 4H, 8^2^-CH_3_) ppm. ^13^C{^1^H} NMR (101 MHz, pyridine-*d*_5_): δ = 196.0, 176.1, 172.8, 162.4, 155.7, 151.7, 149.5, 145.8, 142.0, 138.7, 137.0, 136.7, 136.3, 132.5, 131.7, 130.0, 128.9, 123.0, 107.4, 104.9, 97.9, 94.5, 52.7, 50.7, 48.8, 32.3, 31.1, 23.7, 20.0, 18.1, 12.5, 12.3, 11.6 ppm. Maldi-ToF MS: calcd for C_33_H_34_N_4_O_3_ *m/z* 534.263, found 534.290. UV-Visible (THF): λ_max_ (ε) 414 (77500), 507 (8200), 536 (6600), 310 (5300), 668 (33600) nm.

**M3**: **Chlorin-1** (117 mg, 0.180 mmol) was dissolved in trifluoroacetic acid (6.5 mL). The green solution was stirred for 3 h at room temperature and then, poured into water. The organic layer was extracted with CH_2_Cl_2_, dried over MgSO_4_, filtered and concentrated under reduced pressure. The brown residue was purified by column chromatography on silica gel (CH_2_Cl_2_/MeOH, 19:1) to give the title compound as a black solid (68 mg, 64%). ^1^H-NMR (600 MHz, CDCl_3_/pyridine-*d*_5_): δ = 9.41 (s, 1H, 10-H), 9.37 (s, 1H, 5-H), 9.03 (d, 1H, ^3^*J*_H-H_ = 16.1 Hz, 3^2^-CH), 8.56 (s, 1H, 20-H), 7.04 (d, 1H, ^3^*J*_H-H_ = 16.1 Hz, 3^1^-CH), 5.17, 5.01 (d, each 1H, ^3^*J*_H-H_ = 19.2 Hz, 13^2^-CH_2_), 4.40, 4.20 (m, each 1H, 17-, and 18-H), 3.54, 3.48, 3.36, 3.10 (s, each 3H, 2^1^-, 7^1^-, and 12^1^-CH_3_, 17^2^-COOMe), 3.53 (m, 2H, 8^1^-CH_2_), 2.60, 2.50 (m, each 1H, 17^2^-CH_2_), 2.20 (m, 2H, 17^1^-CH_2_), 1.72 (d, 3H, *J* = 7.5 Hz, 18^1^-CH_3_), 1.57 (t, 3H, ^3^*J*_H-H_ = 7.7 Hz, 8^2^-CH_3_), 0.08, −1.97 (s, each 1H, NH) ppm. ^13^C{^1^H} NMR (151 MHz, CDCl_3_/Pyr): δ = 195.6, 173.1, 170.7, 169.1, 160.7, 154.7, 151.0, 149.4, 148.5, 144.7, 140.3, 138.1, 136.3, 135.5, 135.4, 133.5, 131.9, 130.7, 128.5, 126.3, 123.2, 106.2, 103.6, 96.7, 93.5, 51.6, 51.3, 49.6, 47.8, 30.7, 29.6, 22.9, 19.2, 17.2, 12.4, 11.8, 11.0 ppm. ESI-ToF HR-MS: calcd for C_35_H_36_N_4_O_5_ [M + H]^+^ *m/z* 593.2841, found 593.2845. UV-Visible (THF): λ_max_ (ε) 417 (33000), 513 (4600), 545 (3600), 621 (2700), 681 (17000) nm.

**M5**: To a solution of methyl pyropheophorbide-*d* (200 mg, 0.363 mmol) and cyanoacetic acid (6.18 g, 72.6 mmol) in THF (20 mL) was added ammonium acetate (2.80 g, 36.3 mmol). The mixture was refluxed for 1 h, poured into water and extracted with CH_2_Cl_2_. The extract was dried over MgSO_4_, filtered, and concentrated under vacuum. The residue was then recrystallized from a CH_2_Cl_2_/*n*-pentane mixture to give the title compound as a dark solid (145 mg, 65%). ^1^H NMR (400 MHz, DMSO-*d*_6_): δ = 9.62, 9.32, 9.13, 8.62 (s, each 1H, 5-, 10-, and 20-H, 3^1^-CH), 5.14, 5.06 (d, each 1H, ^3^*J*_H-H_ = 19.9 Hz, 13^1^-CH_2_), 5.06, 4.54 (m, each 1H, 17-, and 18-H), 3.75 (m, 2H, 8^1^-CH_2_), 3.59, 3.53, 3.31, 3.23 (s, each 3H, 2^1^-, 7^1^-, and 12^1^-CH_3_, 17^2^-COOMe), 2.60, 2.24 (m, each 2H, 17^1^-, and 17^2^-CH_2_), 1.77 (d, 3H, ^3^*J*_H-H_ = 7.5 Hz, 18^1^-CH_3_), 1.67 (t, 3H, 3*J*_H-H_ = 7.7 Hz, 8^2^-CH_3_) ppm. ^13^C{^1^H} NMR (101 MHz, DMSO-*d*_6_**):** δ = 195.2, 176.5, 172.9, 167.7, 163.2, 160.1, 156.6, 152.0, 149.8, 147.1, 145.3, 145.0, 143.0, 142.7, 137.2, 135.1, 133.1, 132.9, 131.6, 120.5, 119.1, 105.3, 105.1, 98.4, 92.7, 66.4, 66.2, 66.0, 65.7, 65.5, 51.0, 50.1, 47.7, 47.6, 29.9, 29.2, 24.0, 23.8, 23.6, 23.5, 23.4, 23.0, 18.5, 17.2, 13.2, 12.0, 10.4 ppm. ESI-ToF HR-MS: calcd for C_36_H_36_N_5_O_5_ [M + H]^+^ *m/z* 618.2716, found 618.2717. UV-Visible (THF): λ_max_ (ε) 409 (33000), 515 (5000), 550 (4000), 636 (4500), 688 (13000) nm.

### 3.3. General Procedure for the Zinc Metalation to Obtain Dyes ***M2***, ***M4*** and ***M6***

A saturated methanol solution of zinc acetate dihydrate (Zn(OAc)_2_ 2H_2_O; 10 mL) was added to a solution of metal-free chlorin (50 mg) in THF (50 mL). After stirring overnight, the solution turned from brown to green. The mixture was filtered off and the filtrate was concentrated under vacuum. The crude material was then recrystallized from a CH_2_Cl_2_/*n*-pentane mixture to afford the desired zinc complex.

**M2**: (80%). ^1^H-NMR (400 MHz, Pyridine-*d*_5_): δ = 9.84, 9.69, 8.83 (each s, each 1H, 5-, 10-, and 20-H), 8.19 (dd, ^3^*J*_H-H_ = 17.8, 11.6 Hz, 1H, 3^1^-H), 6.35, 6.15 (m, each 1H, 3^2^-CH_2_), 5.56, 4.61 (m, each 1H,13^2^-CH_2_), 4.61, 4.48 (m, each 1H, 17-, and 18-H), 3.70 (m, 5H, 8^1^-CH_2_, and 12^1^-CH_3_), 3.36 (2^1^-CH_3_), 3.22 (s, 3H, 7^1^-CH_3_), 2.93, 2.73 (m, each 2H, 17^1^-, and 17^2^-CH_2_), 1.85 (d, ^3^*J*_H-H_ = 7.2 Hz, 3H, 18^1^-CH_3_), 1.67 (t, ^3^*J*_H-H_ = 7.6 Hz, 4H, 82-CH_3_) ppm. MALDI-ToF MS: calcd for C_33_H_32_N_4_O_3_Zn *m/z* 596.177, found 596.180. UV-Visible (THF): λ_max_ (ε) 405 (23800), 427 (39500), 570 (2500), 609 (4400), 657 (22000) nm.

**M4**: (87%). ^1^H-NMR (400 MHz, CDCl_3_/Pyridine-*d*_5_): δ = 9.41 (s, 1H, 10-H), 9.37 (s, 1H, 5-H), 9.03 (d, 1H, ^3^*J*_H-H_ = 16.1 Hz, 3^2^-CH), 8.56 (s, 1H, 20-H), 7.04 (d, 1H, ^3^*J*_H-H_ = 16.1 Hz, 3^1^-CH), 5.17, 5.01 (d, each 1H, ^3^*J*_H-H_ = 19.2 Hz, 13^2^-CH_2_), 4.40, 4.20 (m, each 1H, 17-, and 18-H), 3.54, 3.48, 3.36, 3.10 (s, each 3H, 2^1^-, 7^1^-, and 12^1^-CH_3_, 17^2^-COOMe), 3.53 (m, 2H, 8^1^-CH_2_), 2.60, 2.50 (m, each 1H, 17^2^-CH_2_), 2.20 (m, 2H, 17^1^-CH_2_), 1.72 (d, 3H, ^3^*J*_H-H_ = 7.5 Hz, 18^1^-CH_3_), 1.57 (t, 3H, ^3^*J*_H-H_ = 7.7 Hz, 8^2^-CH_3_) ppm. ESI-ToF HR-MS: calcd for C_35_H_34_N_4_O_5_Zn [M + H]^+^ *m/z* 655.1899, found 655.1901. UV-Visible (THF): λ_max_ (ε) 414 (37600), 436 (54500), 531 (3500), 576 (4900), 621 (7900), 671 (40100) nm.

**M6**: (78%). ^1^H-NMR (400 MHz, DMSO-*d*_6_): δ = 9.62, 9.13, 8.62 (s, 1H each, 5-, 10-, 15-, and 20-H), 9.32 (s, 1H, 3′-H), 5.14, 5.05 (d, 1H each, ^3^*J*_H-H_ = 19.9 Hz, 15′-H), 4.55 (dd, 1H, ^3^*J*_H-H_ = 7.4, 2.5 Hz, 18-H), 4.28 (d, 1H, ^3^*J*_H-H_ = 8.2 Hz, 17-H), 3.59, 3.53, 3.31, 3.23 (s, 3H each), 2.60, 2.24 (m, 4H), 1.76 (d, 3H, ^3^*J*_H-H_ = 7.7 Hz, 18^1^-CH_3_), 1.65 (t, 3H, ^3^*J*_H-H_ = 7.6 Hz, 8^2^-CH_3_) ppm. ESI-ToF HR-MS: calcd for C_36_H_35_N_5_O_5_Zn [M + H]^+^ *m/z* 680.1852, found 680.1851. UV-Visible (THF): λ_max_ (ε) 434 (48300), 532 (3800), 579 (4700), 625 (7900), 670 (36800) nm.

### 3.4. DFT Calculations

The ground-state geometry and electronic structure of all dyes and a large TiO_2_ cluster (representative of the bulk) were obtained at the density functional theory (DFT) level using the B3LYP hybrid functional and the 6-311G(d,p) basis set. All calculations were performed using Gaussian09 [57]. Solvent effects were taken into account by employing the polarizable continuum model (PCM) using the chloroform solvent as a proxy for the dielectric effects in the DSSC device.

### 3.5. Fabrication of Solar Cells

FTO-coated conducting glass substrates were cleaned by an ultrasonic treatment with water, acetone and isopropanol for 10 min each before being treated for 5 min by UV-ozone. A compact layer of TiO_2_ was deposited by spray-pyrolysis at 450 °C from a solution of titanium tetra-isopropoxide and acetylacetone in ethanol following by annealing at 450 °C for 20 min (Film thickness 200 nm). A nanoporous TiO_2_ layer was applied by spin-coating from a solution of a commercial TiO_2_ paste (DSL 18NRT, Dyesol) in ethanol containing nano-sized anatase particles (Film thickness 1.6 μm). The layers were then gradually annealed from 250 to 500 °C over 45 min. The substrates were then treated in a 0.02 M TiCl_4_ aqueous solution for 2 h, rinsed with water and annealed at 450 °C for 45 min. The electrodes were finally immersed in dye solutions that may hold chenodeoxycholic acid as co-adsorbent for 15 h in the dark. After rinsing the electrodes, the hole conductor layer was deposited by spin-coating from a spiro-OMeTAD (Solaronix) solution in chlorobenzene (180 mg.mL^−1^) containing tert-butylpyridine and LiTFSI as additives, following a reported recipe [45]. Gold top electrodes were finally evaporated under vacuum (10^−6^ mbar) using shadow masks that define two active areas per substrate (around 0.15 cm^2^ each).

### 3.6. Characterization of Solar Cells

The J–V measurements, in the dark and under illumination, were performed in air using a Keithley model 2400 digital source meter by applying independent external voltage to the cell and by measuring the photogenerated current out from the cell. The spectral mismatch between the emission solar simulator (Newport 1600W) and the global AM1.5G solar spectrum was corrected using a mismatch factor and the solar simulator irradiance was adjusted accordingly using a certified silicon reference cell in order to achieve an equivalent AM1.5G irradiance of one sun (100 mW·cm^−2^) on the test cells. The incident photon to current efficiency (IPCE) was estimated using a monochromated 75 W xenon lamp (Newport). The photocurrent, measured in static regime by a calibrated picoammeter (Keithley 485), was compared to the calibration current response recorded using a certified calibrated photodiode (Newport). All data shown in this work were the average values of at least four parallel tests.

### 3.7. Fabrication of Solar Cells for EPR Experiments

FTO slides were washed and sonicated five times for 15 min each in water, ethanol, twice in isopropanol and finally, analytical grade ethanol. FTO–TiO_2_ (18 NRT Solaronix) films were prepared by “doctor blading” (uniformly spreading TiO_2_ paste on a pre-defined area of 1.25 cm^2^ on FTO glass with a glass bar). The FTO electrode was then left for 30 min on an oven at 450 °C in order to sinter the anatase crystals. The dye adsorption on FTO–TiO_2_ film electrodes was carried out by heating the electrodes for 10 min at 75 °C on a hot plate, followed by immersion into a single dye solution in 50% acetonitrile 50% *t*BuOH for a given time of 16 h. The DSSCs were electrically characterized by the use of a solar simulator (Newport model 96,000 Oriel), equipped with a 150 W ozone free xenon lamp, and a digital multimeter (Keithley 2600A) connected to a computer that used dedicated software for test running.

### 3.8. EPR Characterization of Solar Cells

Before performing the EPR analysis of the complete DSSC, it was necessary to measure the solar cell electrical performance in the absence and the presence of the spin probe. Indeed, only cells showing well-measurable and reproducible electrical performances (not reported) were tested by EPR. After measuring their electrical functionality, the cells were placed on the EPR cavity where a static magnetic field is applied while a source of microwaves is also applied to the sample. This allows the resonance condition that is necessary to promote the probe (**4C-T**) signal. EPR spectra were recorded with an EMX-Bruker spectrometer operating at the X band (9.5 GHz) and interfaced with a PC (software from Bruker for handling and recording the EPR spectra). The EPR spectra were simulated with the NLSL software [58].

### 3.9. Transient Photovoltage Decay Mesurements

Transient photovoltage measurements were done on the cells illuminated by a bias light provided by white LED of variable intensity [59]. An additional light pulse was superimposed using a 550 nm green LED, and the transient electrical response of the cell was recorded in open-circuit conditions using the high impedance input of a digital oscilloscope (Tektronix DPO4032, Tektronix, Bracknell, UK) Transient photovoltage curves were fitted using mono-exponential functions, ensuring a perturbing regime where the transient photogenerated charge density was kept below 1% of the stead-state photogenerated charge density.

## 4. Conclusions

A series of chlorophyll derivatives **M1**–**M6** equipped with either a non-conjugated carboxy or a conjugated acrylic or cyanoacrylic anchoring group was prepared for their use as dye component in all-solid state DSSCs. The use of a co-adsorbent was found to be crucial to improve the device performance by avoiding the chlorin dye aggregation. The solar energy conversion efficiencies of DSSCs bearing the acrylic group (**M3**–**M4**) were larger than those bearing the cyanoacrylic group (**M5**–**M6**). The weakest performances of **M5**–**M6** compared to **M3**–**M4** were attributed to several factors: (i) a faster recombination kinetic at the titania/electrolyte interface, (ii) the position of the LUMO below the TiO_2_ conduction band is not suitable for electron injection, and (iii) a lower amount adsorbed onto the TiO_2_ surface. Nevertheless, although **M1**–**M2** exhibited not suitable features in terms of recombination kinetics and dye adsorption, higher PCEs were obtained which may suggest that **M1**–**M2** molecules have a suitable orientation on the TiO_2_ surface due the larger flexibility of the alkyl spacer, which may lead to improved charge transfer. This question will be addressed in future investigations as well as their long-term stability.

## Figures and Tables

**Table 1:** Spectral characteristics of chlorin derivatives **M1**–**M6** in THF solution and grafted on the TiO_2_ surface.

Compound	Solvent	λ_max_/nm		
		Soret	Q_x_(0,1)/(0,0)	Q_Y_(0,1)/(0,0)
**M1**	THF	414	507/536	610/668
	TiO_2_		520/553	620/673
**M2**	THF	427	524/570	609/657
	TiO_2_		524/541	633/681
**M3**	THF	417	513/545	621/681
	TiO_2_		518/560	632/685
**M4**	THF	436	531/576	621/671
	TiO_2_		524/541	631/681
**M5**	THF	409	515/550	636/688
	TiO_2_		518/560	632/691
**M6**	THF	434	532/579	625/672
	TiO_2_		527/595	635/689

**Table 2:** Photovoltaic parameters of ssDSSCs sensitized with **M2** or **D102** under simulated solar emission (100 mW·cm^−2^, AM1.5G) as a function of the co-adsorbent concentration: short-circuit current density (*J*_SC_), open-circuit voltage (V_OC_); fill factor (FF), and power conversion efficiency (η).

Concentration	V_OC_ (V)	*J*_SC_ (mA·cm^−2^)	FF	η	Best η
0 mM	0.54 ± 0.01	0.73 ± 0.10	47 ± 4	0.18 ± 0.01	0.19
0.3 mM	0.52 ± 0.02	1.27 ± 0.24	36 ± 3	0.23 ± 0.02	0.25
1.5 mM	0.65 ± 0.03	1.73 ± 0.19	48 ± 5	0.53 ± 0.03	0.56
3 mM	0.56 ± 0.02	1.96 ± 0.24	40 ± 0	0.39 ± 0.03	0.42
**D102**	0.76 ± 0.01	10.01 ± 0.19	49 ± 3	3.73 ± 0.26	3.99

**Table 3:** Photovoltaic parameters of ssDSSCs sensitized with chlorins **M1–M6** and **D102** under simulated solar emission (100 mW·cm^−2^, AM 1.5G): short-circuit current density (*J*_SC_), open-circuit voltage (V_OC_), fill factor (FF), and power conversion efficiency (η).

Compound	V_OC_ (V)	*J*_SC_ (mA·cm^−2^)	FF	η	Best η
**M1**	0.54 ± 0.03	1.99 ± 0.27	42 ± 3	0.45 ± 0.15	0.60
**M2**	0.65 ± 0.03	1.73 ± 0.19	48 ± 5	0.53 ± 0.03	0.56
**M3**	0.53 ± 0.05	2.31 ± 0.63	46 ± 4	0.55 ± 0.12	0.67
**M4**	0.54 ± 0.04	1.62 ± 0.20	49 ± 4	0.43 ± 0.08	0.51
**M5**	0.54 ± 0.02	1.16 ± 0.12	54 ± 6	0.34 ± 0.05	0.39
**M6**	0.53 ± 0.02	0.97 ± 0.15	52 ± 4	0.26 ± 0.07	0.33

**Table 4:** Values of the total intensity and the relative percentage for the slow and fast component.

Dye	Total Intensity (Integrated; Arbitrary Unit)	% of Slow Component with Respect to the Fast	% of Slow Component with Respect to the Total
**M1**	64	5	3
**M3**	52	35	18
**M5**	14	69	10
